# FOXC1 overexpression is a marker of poor response to anthracycline-based adjuvant chemotherapy in sporadic triple-negative breast cancer

**DOI:** 10.1007/s00280-017-3319-4

**Published:** 2017-05-10

**Authors:** Y. L. Xu, R. Yao, J. Li, Y. D. Zhou, F. Mao, B. Pan, Q. Sun

**Affiliations:** 1 Department of Breast Surgery, Peking Union Medical College Hospital, Peking Union Medical College, Chinese Academy of Medical Sciences, Beijing, China; 2 Department of Pathology, Peking Union Medical College Hospital, Peking Union Medical College, Chinese Academy of Medical Sciences, Beijing, China

**Keywords:** FOXC1, Anthracycline, Chemosensitivity, Prognosis, TNBC

## Abstract

**Purpose:**

Because of its aggressive characteristics and poor prognosis, triple-negative breast cancer (TNBC) has become a hot topic in cancer research. Chemotherapy is currently the only treatment for patients with TNBC. The transcription factor FOXC1 has been associated with TNBC prognosis, but little is known about its effect on chemosensitivity. The aim of this study was to investigate the effects of FOXC1 on chemosensitivity.

**Methods:**

A case–control study was performed on 25 TNBC patients who experienced relapse and/or metastasis. Another 25 patients without relapse or metastasis were randomly selected as controls. Medical records were reviewed for relevant information, and immunohistochemistry was performed to measure FOXC1 levels. The Kaplan–Meier method and Cox analysis were used to analyze differences in disease-free survival (DFS) and overall survival (OS). The correlation of FOXC1 expression with chemosensitivity was analyzed. Data were analyzed using SPSS 21.0 software, and a *P* value <0.05 was considered to be statistically significant.

**Results:**

In 15 of 22 case patients, FOXC1 was overexpressed, whereas only 8 control patients exhibited FOXC1 overexpression (*P* < 0.05). FOXC1 expression had no correlation with pathological indicators. An anthracycline-based regimen was administered to 21 study patients and 23 control patients. FOXC1 expression was significantly associated with a worse DFS (HR 2.62, 95% CI 1.05–6.50, *P* = 0.038) but presented no correlation with OS (HR 2.53, 95% CI 0.76–8.40, *P* = 0.131) among these 44 patients.

**Conclusions:**

This study shows that FOXC1 is correlated with chemosensitivity to anthracycline and could be used as an indicator of chemosensitivity in sporadic TNBC.

**Electronic supplementary material:**

The online version of this article (doi:10.1007/s00280-017-3319-4) contains supplementary material, which is available to authorized users.

## Introduction

Triple-negative breast cancer (TNBC) is defined by the lack of expression of estrogen receptor (ERα) and progesterone receptor (PR) as well as the absence of overexpression and/or gene amplification of HER2 [1–3]. This subtype of breast cancer accounts for approximately 10–15% of all breast cancers [4–6], and more studies have focused on this subtype because of its aggressive clinical behavior and poor prognosis [5, 7]. Although ERα- and HER2-targeted treatments are used for luminal and HER2-positive breast cancers, respectively, chemotherapy remains the only modality of systemic therapy for TNBC [8]. Several studies have shown that compared to other types of breast cancer, TNBC in general is more sensitive to chemotherapy [9, 10]; however, only a minority of TNBC patients have an excellent outcome after receiving standard chemotherapy. Despite receiving standard cytotoxic chemotherapy, approximately 30–40% of patients with early-stage TNBC develop metastatic disease, eventually succumbing to their cancer [9–11]. These observations suggest that patients with TNBC comprise a heterogeneous group [12] and that it is thus important to identify the subgroup of TNBC patients who may benefit from adjuvant chemotherapy. Several previous reports have shown that FOXC1 is closely correlated with prognosis [2] and has the potential to be a therapeutic target in TNBC [13]. However, there are no reports on the role of FOXC1 in response to chemotherapy in clinical settings.

FOXC1 is a member of the forkhead box (FOX) transcription factor superfamily, which plays important roles in cell growth, survival, differentiation, migration, and longevity [14, 15]. Previously, it has been shown that ectopic overexpression of FOXC1 in breast cancer cell lines induces aggressive phenotypes [2, 16, 17]. Conversely, shRNA knockdown of FOXC1 in breast cancer cell lines with high endogenous levels of FOXC1 led to opposing effects with the loss of aggressive phenotypic features [13]. Another study indicated that FOXC1 demethylation, which results in its overexpression, is closely correlated with chemoresistance in locally advanced breast cancer patients receiving neoadjuvant anthracycline treatment [18]. Therefore, it is important to investigate the effects of FOXC1 on chemosensitivity and to determine whether FOXC1 might be a potential biomarker for regimen selection in TNBC patients.

Jia and his colleagues have shown that FOXC1 may play a role in the degree of malignancy and drug resistance of relapsing invasive ductal carcinoma [19]. However, it is unclear whether overexpression of FOXC1 in sporadic TNBC impacts the patient response to chemotherapy. The goal of this study was to investigate the prognostic significance of FOXC1 expression in early-stage TNBC patients treated with standard chemotherapy and the effect of FOXC1 overexpression on the chemotherapeutic response in triple-negative breast cancer patients.

## Materials and methods

### Ethics statement

This study was approved by the Institutional Review Board (IRB) at the Peking Union Medical College Hospital, Beijing, China. Fifty patients who were pathologically diagnosed with TNBC and signed informed consent upon surgical intervention at our center were enrolled in the study.

### Patients and study design

A total of 3154 consecutive patients with operable primary breast cancer were treated at Peking Union Medical College Hospital from December 2007 to April 2012. Of these patients, 253 (approximately 8%) were TNBC according to pathology (additional data are given in online Fig. S1). Two hundred and forty-seven TNBC patients received adjuvant chemotherapy, and with a median follow-up time of 32 months (range 2–68 months), follow-up data were available for 88.67% of patients (219/247). During follow-up, 25 patients developed local recurrence and/or distant metastasis, and 12 patients died of breast cancer. Of 194 TNBC patients without local recurrence or distant metastasis, 25 were randomly selected as controls. Formalin-fixed paraffin-embedded (FFPE) samples from these selected patients were retrieved from the pathology archives. Each tumor specimen was evaluated by two pathologists to confirm the presence of invasive disease, and only samples with >50% invasive cancer were included in the analysis. Archived tissue blocks of 47 patients with adequate invasive cancer were available and comprised the study cohort. None of the patients received neoadjuvant chemotherapy. Demographic and clinical information regarding the pathological stage, breast cancer treatment, outcome, etc., was collected by reviewing the medical record.

### Histopathological analysis: ER/PR/HER2/FOXC1

Triple-negative breast cancer was defined as a negative ER, PR, and HER2 status. Immunohistochemistry (IHC) staining was scored using criteria from published guidelines. Immunohistochemical nuclear staining of less than or equal to 1% was considered a negative result for ER and PR (in accordance with the 2010 ASCO/CAP guidelines). HER2-negative tumors were defined as 0 or 1+ on IHC staining and/or the lack of gene amplification in fluorescence in situ hybridization (FISH) tests (i.e., a ratio less than 2.0). IHC slides from the selected study patients were reviewed by two board-certified, specialty-trained breast pathologists who remained blinded to the clinical data. The ER, PR, and HER2 status at the time of this study were in agreement with the initial diagnosis after surgery.

**Fig. 1:**
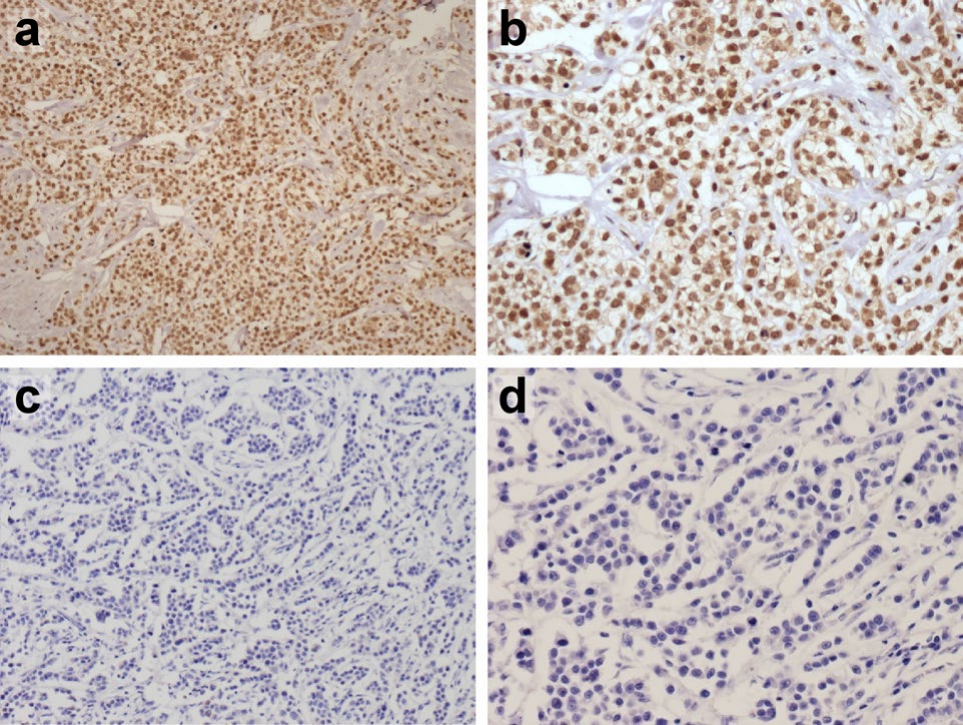
Representative images of FOXC1 staining in human breast tumor. Magnification: **a** FOXC1 positive (×100); **b** FOXC1 positive (×200); **c** FOXC1 negative (×100); **d** FOXC1 negative (×200)

We performed IHC analysis of FOXC1 on whole-tissue sections from archived FFPE tissue blocks for all TNBC patients in our study cohort (rabbit polyclonal IgG FOXC1 antibody, catalog No. LS-B1800, Lifespan Bioscience) [2]. Tissue blocks were sectioned into serial 5-μm-thick tissue sections and subjected to IHC analysis to detect FOXC1. Semi-quantitative analysis was performed by pathologists who scored the staining intensity (SI) and percentage of positive cells (PP) according to the immunoreactive score (IRS) criteria recommended by Remmele and Stegner [20, 21]. This method evaluates both the percentage of positive cells and the staining intensity of the nuclei, and has been used in analyzing the IHC expression of many proteins, such as FOXC1, CXCL12 [22, 23]. The details are as follows: The intensity of immunoreactivity was recorded as 0 (no staining), 1 (weak staining), 2 (moderate staining), or 3 (strong staining). The percentage of positive cells was recorded on a scale of 0 (no positive cells), 1 (≤10% positive cells), 2 (11–50% positive cells), 3 (51–75% positive cells), or 4 (>75% positive cells). The arithmetic product of the SI and PP was used to determine FOXC1 expression as either negative (score 0–3) or positive (≥4) (Fig. 1).

### Statistical analysis

SPSS 21.0 and GraphPad Prism 5.0 were used for statistical analyses. Data were described as numbers (percentages) or the means and standard deviation. Patient characteristics were compared between groups (presence vs. absence of local recurrence/distant metastasis) by a Chi-square test or Mann–Whitney *U* test as appropriate. The Chi-square test was used to determine the association between clinical/histopathological parameters and FOXC1 expression. The time to recurrence was defined as the date of diagnosis to either the date of local or systemic recurrence, or the last follow-up. Overall survival (OS) was measured from the date of diagnosis to the date of death or the last follow-up. Survival outcomes were estimated according to the Kaplan–Meier method and compared between groups by using the log-rank statistic. Cox regression analysis was used to determine the association of FOXC1 with the risk of recurrence and death after adjusting for other characteristics. *P* values <0.05 were considered to be statistically significant.

## Results

### Study population

This study enrolled 50 patients with stage I–stage III TNBC who underwent definitive surgery at our institution between October 2007 and April 2012. Their tumor specimens were available and identified in our pathology archives. Of these 50 subjects with TNBC, 47 had an adequate tumor specimen available for analysis. Table 1 describes the baseline demographics of the study population, and there were no differences between the two groups except for FOXC1 expression. The median age was 47 years (range 29–82 years). The median primary tumor size according to the pathology reports was 2.4 cm (range 0.7–11 cm), with 89% (42/47) of patients receiving modified radical mastectomy, 6% (3/47) of patients receiving conservative surgery, and the remaining patients undergoing either wide local excision or simple mastectomy. Among 22 of the TNBC patients with recurrence or metastasis, 1 patient had local recurrence, 3 patients had local recurrence and distant metastasis, and 18 patients had distant metastasis; bone metastasis was the most common metastatic event.

**Table 1:** Clinical and pathological characteristics

Characteristics	Total	Case	Control	*P* value
Total	47	22	25	
Age (mean ± SD)	47	49.36 ± 13.37	46.80 ± 8.32	0.38
Menopausal status				0.387
Premenopausal	25	10	15	
Postmenopausal	22	12	10	
Tumor size (cm)				0.421
≤2	20	8	12	
>2	27	14	13	
Number of positive LNs				0.609
Negative	8	3	5	
Positive	39	19	20	
Histological type				1
IDC	45	21	24	
Others	2	1	1	
Histological grade				0.219
Well or moderate	10	3	7	
Poor	37	19	18	
LVI				0.819
Positive	10	5	5	
Negative	37	17	20	
p53 expression				0.391
Positive	29	15	14	
Negative	18	7	11	
Ki-67 (%)				0.849
<14	8	3	5	
≥14	39	19	20	
Surgery				0.55
Modified radical mastectomy	42	19	23	
Breast-conserving surgery	3	2	1	
Wide local excision/simple mastectomy	2	1	1	
AJCC clinical stage				0.804
I	5	1	4	
II	18	10	8	
III	24	11	13	
Chemotherapy				0.629
Anthracycline based	44	21	23	
Others	3	1	2	
Radiotherapy				0.196
Yes	22	12	10	
No	25	10	15	
FOXC1 expression				0.013*
Positive	23	15	8	
Negative	24	7	17	

### FOXC1 has no association with other clinical/histopathological parameters

Semi-quantitative IHC scoring showed that 68% (15/22) of the patients with recurrence or metastasis had FOXC1 overexpression, whereas only 32% (8/25) of the patients without recurrence or metastasis were FOXC1 positive (*P* < 0.05). The clinical and histopathological parameters were compared based on FOXC1 expression. There were no statistically significant associations between FOXC1 expression and age, menopausal status, tumor size, axillary lymph node status, histological type, differentiation, lymphovascular invasion, p53 status, Ki-67 index, or AJCC clinical stages as shown in Table 2. Hence, FOXC1 is an independent histopathological factor.

**Table 2:** Association between clinical/histopathological factors and FOXC1 expression

Characteristics	Total	FOXC1 expression		*P* value
		Positive (*N* = 23)	Negative (*N* = 24)	
Age (mean ± SD)	47	50.57 ± 12.42	45.54 ± 8.85	0.17
Menopausal status				0.564
Premenopausal	25	11	14	
Postmenopausal	22	12	10	
Tumor size (cm)				0.38
≤2	20	8	12	
>2	27	15	12	
Number of positive LNs				0.245
Negative	8	2	6	
Positive	39	21	18	
Histological type				0.976
IDC	45	22	23	
Others	2	1	1	
Histological grade				1
Well and moderate	10	5	5	
Poor	37	18	19	
LVI				0.435
Positive	10	6	4	
Negative	37	17	20	
p53 expression				0.631
Positive	29	15	14	
Negative	18	8	10	
Ki-67 (%)				0.482
<14	8	3	5	
≥14	39	20	19	
AJCC clinical stage				0.309
I	5	1	4	
II	18	9	9	
III	24	13	11	

### FOXC1 is an indicator of poor prognosis

Positive expression of FOXC1 protein was a significant predictor of DFS at a median follow-up of 32 months (range 2–68 months) (additional data are given in online Table S1 and Fig. S2a) based on univariate analysis [hazard ratio (HR) 2.60, 95% confidence interval (CI) 1.11–6.09, *P* = 0.027], but was not a significant predictor of OS (additional data are given in online Table S2 and Fig. S2b). The median DFS was 19 months for the FOXC1-positive triple-negative breast cancer, and 32 months for the FOXC1-negative patients. Other standard clinicopathological factors such as age, menopausal status, tumor size, nodal status, and tumor grade were not significant predictors of either DFS or OS in our study. The prognostic significance of FOXC1 protein expression as an independent predictor of DFS persisted after multivariate analysis (HR 2.83, 95% CI 1.09–7.40, *P* = 0.034), but this analysis showed that FOXC1 expression was not an independent predictor of OS in our study.

### FOXC1 overexpression is an indicator of chemoresistance to anthracycline-based chemotherapy

**Fig. 2:**
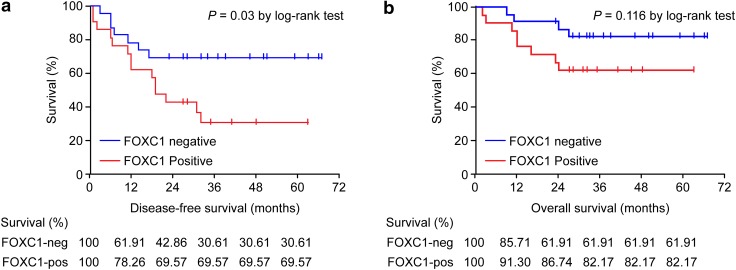
Kaplan–Meier plots of patient survival based on the FOXC1 expression status in patients receiving anthracycline-based adjuvant chemotherapy. **a** DFS. **b** OS. The log-rank test was used to calculate the *P* value

FOXC1 expression was tested for its association with survival by a separate log-rank test in groups based on different adjuvant chemotherapy regimens (additional data are given in online Tables S3 and S4). In the anthracycline-based patient group, breast cancer-specific DFS was significantly improved in patients without FOXC1 protein overexpression (*P* = 0.03, Fig. 2a). However, FOXC1 overexpression was not significantly correlated with breast cancer-specific OS in this patient group (*P* = 0.116, Fig. 2b). However, a trend for improved survival was observed in other patient groups without FOXC1 expression (additional data are given in online Tables S3 and S4). These findings regarding FOXC1 expression indicate a treatment-specific effect on survival in patients receiving anthracycline-based chemotherapy.

Survival analysis in the anthracycline-treated cohort based on the log-rank test indicated that FOXC1 expression (*P* = 0.038) and tumor size (*P* = 0.006) could slightly better differentiate between the two survival groups in the analyzed sample collection, whereas differences in groups classified by menopausal status, nodal status, and tumor differentiation did not reach statistical significance (Tables 3, 4).

**Table 3:** Relationship between DFS and clinicopathological features in 44 patients treated with anthracyclines in adjuvant settings

Characteristics	Total	Recurrence or metastasis		*P* value
		Yes	No	
Age (mean ± SD)	44	49.76 ± 13.75	46.09 ± 8.10	0.192
Menopausal status				0.251
Premenopausal	24	9	15	
Postmenopausal	20	12	8	
Tumor size (cm)				0.006*
≤2	17	7	10	
>2	27	14	13	
Number of positive LNs				0.648
Negative	8	3	5	
Positive	36	18	18	
Histological grade				0.092
Well and moderate	9	2	7	
Poor	35	19	16	
LVI				0.752
Positive	10	5	5	
Negative	34	16	18	
p53 expression				0.55
Positive	27	14	13	
Negative	17	7	10	
Ki-67 (%)				0.469
<14	7	3	4	
≥14	37	18	19	
AJCC clinical stage				0.53
I	5	1	4	
II	18	10	8	
III	21	10	11	
FOXC1 expression				0.038*
Positive	21	14	7	
Negative	23	7	16

**Table 4:** Relationship between OS and clinicopathological features in 44 patients treated with anthracyclines in adjuvant settings

Characteristics	Total	Failure event		*P* value
		Yes	No	
Age (mean ± SD)	44	48.17 ± 13.57	47.72 ± 11.08	0.768
Menopausal status				0.799
Premenopausal	24	7	17	
Postmenopausal	20	5	15	
Tumor size (cm)				0.012*
≤2	17	4	13	
>2	27	8	19	
Number of positive LNs				0.851
Negative	8	2	6	
Positive	36	10	26	
Histological grade				0.257
Well and moderate	9	1	8	
Poor	35	11	24	
LVI				0.259
Positive	10	4	6	
Negative	34	8	26	
p53 expression				0.507
Positive	27	7	20	
Negative	17	5	12	
Ki-67 (%)				0.381
<14	7	2	5	
≥14	37	10	27	
AJCC clinical stage				0.184
I	5	1	4	
II	18	3	25	
III	21	8	3	
FOXC1 expression				0.131
Positive	21	8	13	
Negative	23	4	19	

To identify significant parameters contributing to the observed difference in DFS, Cox regression analysis was performed. The hazard ratio for each of the contributing factors was either estimated separately (univariate analysis) or modeled together (multivariate analysis). Univariate analysis identified tumor size and FOXC1 overexpression as significant predictors of DFS (Table 5). To investigate whether tumor size and FOXC1 expression were independent prognostic markers, we performed a multivariate analysis which showed that the patients in this study with larger tumors (HR 1.50, 95% CI 1.12–1.99, *P* = 0.006) and FOXC1 overexpression (HR 2.58, 95% CI 1.04–6.42, *P* = 0.041) had a higher risk of suffering from local recurrence and/or distant metastasis compared with patients with smaller tumors and/or no to low FOXC1 expression. However, both the univariate and multivariate analyses showed that only tumor size was a significant predictor of OS (additional data are given in online Table S5), but that FOXC1 overexpression was not.

**Table 5:** Univariate and multivariate analyses of parameters that predict DFS in TNBC patients treated with anthracyclines in adjuvant settings by Cox regression analysis

Prognostic factor	HR	95% CI	*P* value
Univariate analysis			
FOXC1 expression	2.62	1.05–6.50	0.038*
Patient age	1.03	0.98–1.08	0.192
Menopausal status	1.16	0.37–3.66	0.251
Tumor size	1.53	1.13–2.06	0.006*
Tumor grade	4.11	0.95–17.7	0.092
LVI	1.18	0.43–3.21	0.752
Lymph node status	1.4	0.59–3.30	0.648
p53	0.68	0.22–2.11	0.55
Ki-67	1.39	0.57–3.35	0.469
Multivariate analysis			
FOXC1 expression	2.58	1.04–6.42	0.041
Tumor size	1.5	1.12–1.99	0.006

## Discussion

It is well established that patients with TNBC have worse outcomes than patients with other breast cancer subtypes. Currently, chemotherapy is the only systemic treatment for TNBC patients; however, some patients with a subclassification of TNBC are not sensitive to chemotherapy. Therefore, a predictive marker must be identified that can discern the sensitivity of TNBC patients to chemotherapy and can avoid overtreatment of the resistant subgroup. Our results suggest that FOXC1 expression in sporadic TNBC predicts poor prognosis in patients receiving anthracycline-based chemotherapy.

Recently, the transcriptional factor FOXC1 has received substantial attention, especially regarding its correlation with chemosensitivity. Dejeux et al. [18] investigated the methylation status of the promoter regions of FOXC1 in doxorubicin-treated locally advanced primary breast tumors. Although FOXC1 with methylated promoters was almost exclusively not expressed, the expression and methylation status of FOXC1 were not significantly correlated, as FOXC1 was already silenced in most tumors independent of its methylation status. However, as basal-like breast tumors generally showed a lower degree of methylation than the other subtypes, it is reasonable to expect that FOXC1 overexpression is more common in TNBC. A significant difference in patient survival between methylated and unmethylated samples was confirmed as patients with an unmethylated promoter region had lower survival rates. Our results are in accordance with this report and indicate that FOXC1 expression has the potential to predict chemosensitivity in anthracycline-based chemotherapy.

In the multivariate analysis by Cox regression, only tumor size and FOXC1 were significant factors related to DFS, whereas common pathological factors [such as age, nodal status, differentiation, and lymphovascular invasion (LVI)] were not. However, in most previous studies, at least the nodal status, differentiation, and LVI were significant factors related to survival. As FOXC1 overexpression is a factor that precedes tumor invasiveness, changes in FOXC1 expression might affect those aforementioned insignificant factors. However, the mechanism of FOXC1’s correlation with anthracycline resistance requires further investigation. Some studies have focused on pathways, including FOXC1-related signaling. Cui et al. have shown that FOXC1 is involved in pathways relevant to EGFR [13], NF-κB [24], and Hedgehog [25] signaling. Whether FOXC1 affects chemosensitivity through these pathways warrants further study.

Screening TNBC patients to identify those who might be resistant to chemotherapy is a reasonable approach. Currently, routine clinical and pathological variables do not clearly identify TNBC patients who are likely to develop recurrence following standard chemotherapy. Many researchers have focused on this problem, with particular attention on in vitro chemosensitivity assays and the identification/development of new biomarkers. As our study showed FOXC1 as a promising biomarker in predicting poor sensitivity to anthracycline-based chemotherapy and detecting FOXC1 expression status is easy via IHC, FOXC1 should be included in clinical routine pathology tests. Determining FOXC1 expression levels by using IHC is relatively inexpensive and can be performed on FFPE tissue; therefore, the implementation of this test has the potential for ease of application in clinical settings.

Although important, our study has several limitations. Notably, the results are subject to bias due to the retrospective nature of the study and its small sample size. We also believe that the small sample size and relatively short follow-up time limited our ability to adequately evaluate the correlation of FOXC1 with OS as well as with other chemotherapeutics; therefore, further study in a larger sample size is warranted. However, our study adds the prognostic impact of FOXC1 expression in patients with TNBC to the existing literature, and this is the first study to evaluate the prognostic impact of FOXC1 in the context of modern chemotherapy. It is impossible to evaluate chemosensitivity and resistance in adjuvant settings as no target is available. Hence, the sensitivity and resistance are extrapolated according to the patient’s survival status. Our study is the first to use FOXC1 to predict the chemosensitivity of TNBC subtypes to anthracycline in adjuvant settings. These results shed light on understanding the value of FOXC1 in predicting the chemosensitivity of TNBC subtypes.

All in all, anthracycline is an important component of chemotherapy regimens for breast cancer treatment and appears to be effective, particularly among patients with early-stage TNBC. However, the relative efficacy of anthracycline on TNBC may be impacted by the expression levels of FOXC1. These data imply that anthracycline-based chemotherapy may not be optimal for patients with tumors that overexpress FOXC1.

Taken together, the present study showed that nearly half of TNBC patients have tumors in which FOXC1 is overexpressed; this discrepancy has the potential to identify a significant percentage of TNBC patients who might have suboptimal outcomes with anthracycline-based standard chemotherapy. Additional research regarding the mechanism of how FOXC1 affects chemotherapeutic efficacy and FOXC1 is warranted to supply further evidence in selecting appropriate chemotherapeutics for breast cancer patients in clinical settings.

## Electronic supplementary material

Below is the link to the electronic supplementary material.

Supplementary material 1 (DOC 865 kb)
