# Supplementary material for: FOXC1 overexpression is a marker of poor response to anthracycline-based adjuvant chemotherapy in sporadic triple-negative breast cancer
Source: Cancer Chemother Pharmacol. 2017 May 10;79(6):1205–13. doi: 10.1007/s00280-017-3319-4 (PMC5438824; doi:10.1007/s00280-017-3319-4)
Supplement: Supplementary file 1 — Supplementary material 1 (DOC 865 kb) [file 280_2017_3319_MOESM1_ESM.doc]

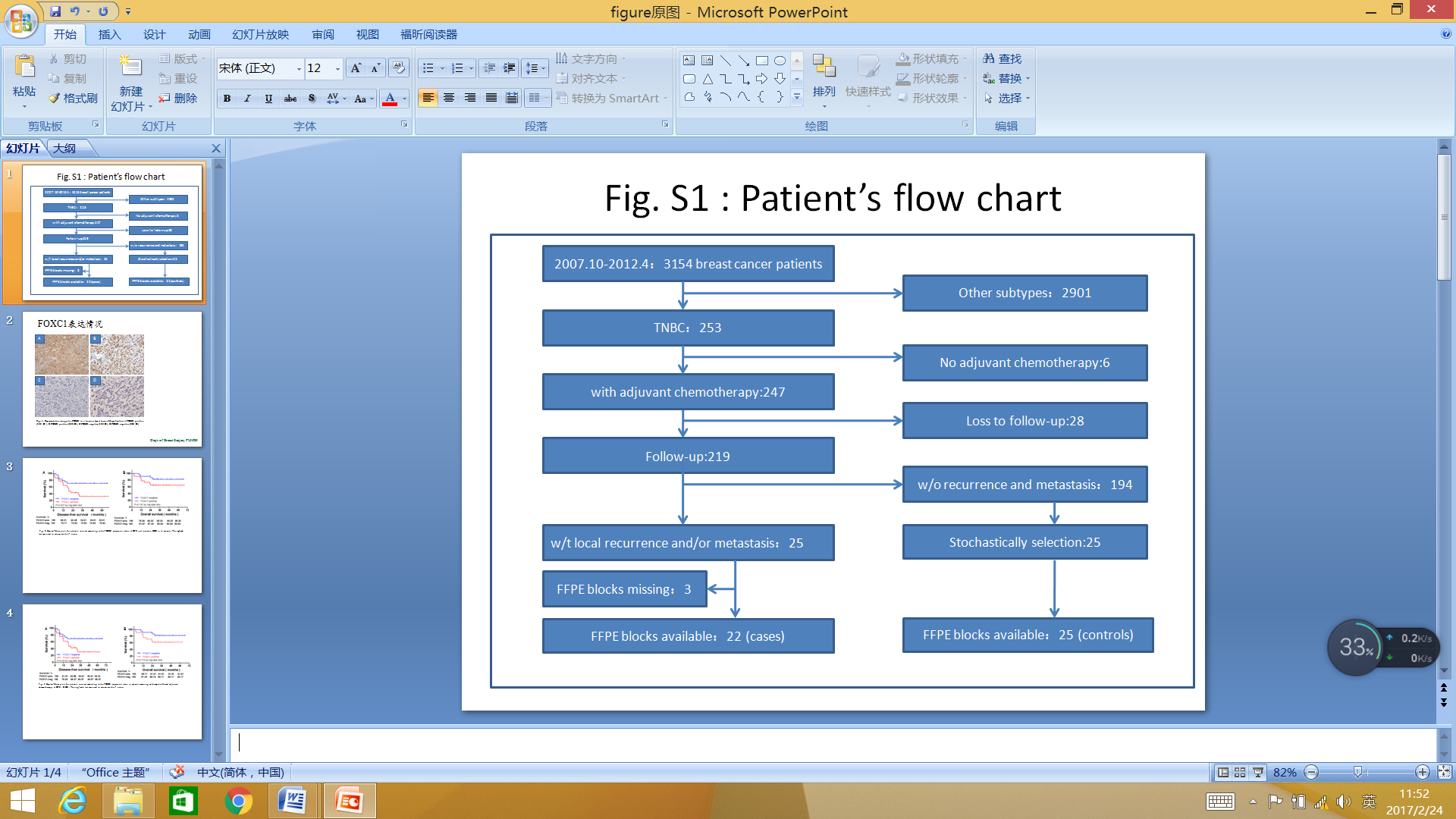


Table S１Relationshop between DFS and clinicopathological features in 47 patients

| Characteristics | Total | Recurrence or Metastasis  Yes No | | P value |
| --- | --- | --- | --- | --- |
| Age (mean±SD) | 47 | 49.36±13.37 | 46.80±8.32 | 0.192 |
| Menopausal status |  |  |  | 0.468 |
| premenopausal  postmenopausal | 25  22 | 10  12 | 15  10 |  |
| Tumor size (cm) |  |  |  | 0.746 |
| ≤2  ＞2 | 20  27 | 8  14 | 12  13 |  |
| Number of positive LNs |  |  |  | 0.694 |
| negative  positive | 8  39 | 3  19 | 5  20 |  |
| Histologic grade |  |  |  | 0.335 |
| Well and moderate  poor | 10  37 | 3  19 | 7  18 |  |
| LVI |  |  |  | 0.689 |
| positive  negative | 10  37 | 5  17 | 5  20 |  |
| P53 expression |  |  |  | 0.378 |
| positive  negative | 29  18 | 15  7 | 14  11 |  |
| Ki-67 (%) |  |  |  | 0.613 |
| ＜14  ≥14 | 8  39 | 3  19 | 5  20 |  |
| AJCC clinical stage |  |  |  | 0.430 |
| Ⅰ  Ⅱ  Ⅲ | 5  18  24 | 1  10  11 | 4  8  13 |  |
| FOXC1 expression |  |  |  | 0.027* |
| Positive  negative | 23  24 | 15  7 | 8  17 |  |

Table S２Relationship between OS and clinicopathological features in 47 patients

| Characteristics | Total | Failure event  Yes No | | P value |
| --- | --- | --- | --- | --- |
| Age (mean±SD) | 47 | 48.17±13.37 | 47.94±10.92 | 0.828 |
| Menopausal status |  |  |  | 0.718 |
| premenopausal  postmenopausal | 25  22 | 7  5 | 18  17 |  |
| Tumor size (cm) |  |  |  | 0.099 |
| ≤2  ＞2 | 20  27 | 5  7 | 15  20 |  |
| Number of positive LNs |  |  |  | 0.947 |
| negative  positive | 8  39 | 2  10 | 6  29 |  |
| Histologic grade |  |  |  | 0.298 |
| Well and moderate  poor | 10  37 | 1  11 | 9  26 |  |
| LVI |  |  |  | 0.200 |
| positive  negative | 10  37 | 4  8 | 6  29 |  |
| P53 expression |  |  |  | 0.439 |
| positive  negative | 29  18 | 6  6 | 23  12 |  |
| Ki-67 (%) |  |  |  | 0.983 |
| ＜14  ≥14 | 8  39 | 2  10 | 6  29 |  |
| AJCC clinical stage |  |  |  | 0.275 |
| Ⅰ  Ⅱ  Ⅲ | 5  18  24 | 1  3  8 | 4  15  16 |  |
| FOXC1 expression |  |  |  | 0.147 |
| Positive  negative | 23  24 | 8  4 | 15  20 |  |

Table S３FOXC1’S correlation with DFS in groups based on different chemotherapy regimens

| Chemotherapy regimen | FOXC1 | Total | Recurrence or metastasis  Yes No | | χ2 | *P*-value |
| --- | --- | --- | --- | --- | --- | --- |
| TA:36 | positive  negative | 16  20 | 10  7 | 6  13 | 1.32 | 0.25 |
| A:44 | positive  negative | 21  23 | 14  7 | 7  16 | 3.96 | 0.03* |
| T:39 | positive  negative | 18  21 | 11  7 | 7  14 | 1.51 | 0.22 |
| C:29 | positive  negative | 14  15 | 8  4 | 6  11 | 2.34 | 0.13 |
| CA:25 | positive  negative | 12  13 | 7  4 | 5  9 | 1.61 | 0.20 |

Table S４ FOXC1’S correlation with OS in groups based on different chemotherapy regimens

| Chemotherapy regimen | FOXC1 | Total | Recurrence or metastasis  Yes No | | χ2 | *P*-value |
| --- | --- | --- | --- | --- | --- | --- |
| TA:36 | positive  negative | 16  20 | 4  4 | 12  16 | 0.11 | 0.74 |
| A:44 | positive  negative | 21  23 | 8  4 | 13  19 | 0.56 | 0.13 |
| T:39 | positive  negative | 18  21 | 4  4 | 14  17 | 0.006 | 0.94 |
| C:29 | positive  negative | 14  15 | 4  2 | 10  13 | 1.04 | 0.31 |
| CA:25 | positive  negative | 12  13 | 4  2 | 8  11 | 1.12 | 0.29 |


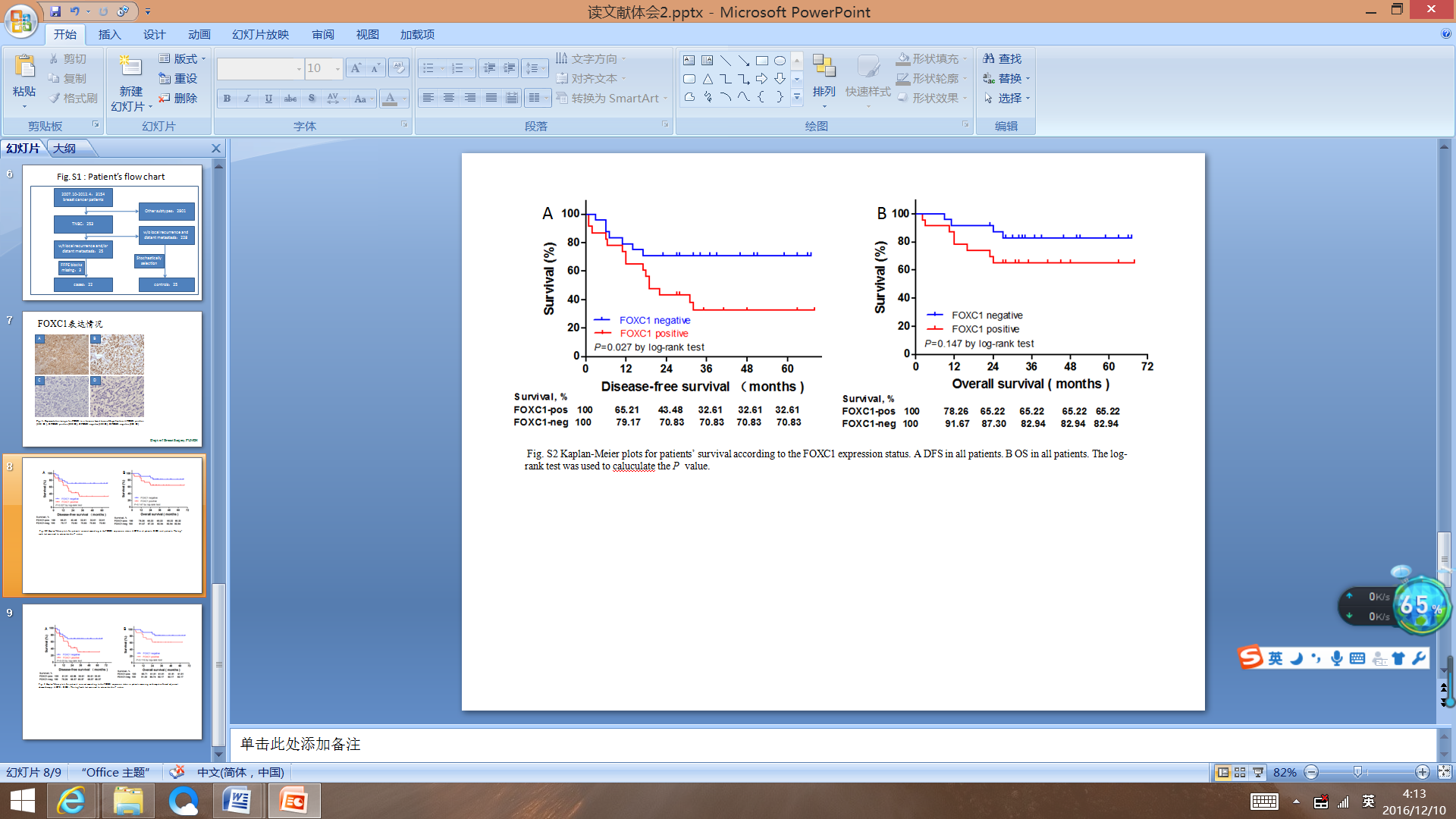


Table S５ Uni- and Multivariate analysis of parameters that predict OS in TNBC using anthracyclines in adjuvant settings by Cox regression analysis

| Prognostic factor | HR | 95%CI～ | *P*-value |
| --- | --- | --- | --- |
| Univariate analysis |  |  |  |
| FOXC1 expression  Patient age  Menopausal status  Tumor size  Tumor grade  LVI  Lymph node status  P53  Ki-67 | 2.53  1.01  1.16  1.64  0.31  2.00  1.16  0.68  1.71 | 0.76～8.40  0.95～1.07  0.37～3.66  1.12～2.40  0.04～2.37  0.60～6.64  0.25～5.28  0.22～2.11  0.51～5.68 | 0.13  0.77  0.80  0.01*  0.26  0.26  0.85  0.51  0.38 |
| Multivatiate analysis |  |  |  |
| FOXC1 expression  Tumor size | 2.55  1.62 | 0.76～8.54  1.12～2.36 | 0.13  0.01* |

Table S6 More detail results of IHC according to the criteria

| No. | Percentage of Positive cells (PP) (%) | Scale of PP | Scale of Staining Intensity (SI) | Arithmetic Product of PP and SI scale | FOXC1 expression |
| --- | --- | --- | --- | --- | --- |
| 1 | 60 | 3 | 1 | 3 | negative |
| 2 | 0 | 0 | 0 | 0 | negative |
| 3 | ＞75 | 4 | 3 | 12 | positive |
| 4 | 75 | 4 | 1 | 4 | positive |
| 5 | ＞75 | 4 | 2 | 8 | positive |
| 6 | 75 | 4 | 2 | 8 | positive |
| 7 | ＞75 | 4 | 2 | 8 | positive |
| 8 | 10 | 2 | 3 | 6 | positive |
| 9 | 0 | 0 | 0 | 0 | negative |
| 10 | 0 | 0 | 0 | 0 | negative |
| 11 | 60 | 3 | 1 | 3 | negative |
| 12 | ＞75 | 4 | 3 | 12 | positive |
| 13 | 30 | 2 | 2 | 4 | positive |
| 14 | 10 | 2 | 3 | 6 | positive |
| 15 | 75 | 4 | 2 | 8 | positive |
| 16 | 50 | 3 | 3 | 9 | positive |
| 17 | 0 | 0 | 0 | 0 | negative |
| 18 | 0 | 0 | 0 | 0 | negative |
| 19 | 10 | 2 | 3 | 6 | positive |
| 20 | ＞75 | 4 | 1 | 4 | positive |
| 21 | 30 | 2 | 2 | 4 | positive |
| 22 | ＞75 | 4 | 1 | 4 | positive |
| 23 | 30 | 2 | 2 | 4 | positive |
| 24 | 10 | 2 | 1 | 2 | negative |
| 25 | 60 | 3 | 2 | 6 | positive |
| 26 | 60 | 3 | 2 | 6 | positive |
| 27 | 60 | 3 | 2 | 6 | positive |
| 28 | 0 | 0 | 0 | 0 | negative |
| 29 | 75 | 3 | 1 | 3 | negative |
| 30 | 0 | 0 | 0 | 0 | negative |
| 31 | 30 | 2 | 2 | 4 | positive |
| 32 | 15 | 2 | 2 | 4 | positive |
| 33 | 10 | 2 | 1 | 2 | negative |
| 34 | 10 | 2 | 1 | 2 | negative |
| 35 | 30 | 2 | 1 | 2 | negative |
| 36 | 0 | 0 | 0 | 0 | negative |
| 37 | 0 | 0 | 0 | 0 | negative |
| 38 | 0 | 0 | 0 | 0 | negative |
| 39 | 0 | 0 | 0 | 0 | negative |
| 40 | 0 | 0 | 0 | 0 | negative |
| 41 | 0 | 0 | 0 | 0 | negative |
| 42 | 10 | 2 | 1 | 2 | negative |
| 43 | 50 | 2 | 1 | 2 | negative |
| 44 | 0 | 0 | 0 | 0 | negative |
| 45 | ＞75 | 4 | 3 | 12 | positive |
| 46 | 75 | 4 | 2 | 8 | positive |
| 47 | 0 | 0 | 0 | 0 | negative |
